# Fibroblast growth factor-21 enhances mitochondrial functions and increases the activity of PGC-1α in human dopaminergic neurons via Sirtuin-1

**DOI:** 10.1186/2193-1801-3-2

**Published:** 2014-01-02

**Authors:** Johanna Mäkelä, Timofey V Tselykh, Francesca Maiorana, Ove Eriksson, Hai Thi Do, Giuseppa Mudò, Laura T Korhonen, Natale Belluardo, Dan Lindholm

**Affiliations:** Institute of Biomedicine/Biochemistry and Developmental Biology, University of Helsinki, Haartmaninkatu 8, FIN-00290 Helsinki, Finland; Minerva Foundation Institute for Medical Research, Biomedicum-2, FIN-00290 Helsinki, Finland; Department of Experimental Biomedicine and Clinical Neuroscience, Division of Human Physiology, University of Palermo, Corso Tukory 129, I-90134 Palermo, Italy

**Keywords:** FGF21, PGC-1α, SIRT1, Dopaminergic neurons, Mitochondria, Parkinson’s disease

## Abstract

Mitochondrial dysfunctions accompany several neurodegenerative disorders and contribute to disease pathogenesis among others in Parkinson’s disease (PD). Peroxisome proliferator-activated receptor γ coactivator-1α (PGC-1α) is a major regulator of mitochondrial functions and biogenesis, and was suggested as a therapeutic target in PD. PGC-1α is regulated by both transcriptional and posttranslational events involving also the action of growth factors. Fibroblast growth factor-21 (FGF21) is a regulator of glucose and fatty acid metabolism in the body but little is known about its action in the brain. We show here that FGF21 increased the levels and activity of PGC-1α and elevated mitochondrial antioxidants in human dopaminergic cells in culture. The activation of PGC-1α by FGF21 occurred via the NAD^+^-dependent deacetylase Sirtuin-1 (SIRT1) subsequent to an increase in the enzyme, nicotinamide phosphoribosyltransferase (Nampt). FGF21 also enhanced mitochondrial respiratory capacity in human dopaminergic neurons as shown in real-time analyses of living cells. FGF21 is present in the brain including midbrain and is expressed by glial cells in culture. These results show that FGF21 activates PGC-1α and increases mitochondrial efficacy in human dopaminergic neurons suggesting that FGF21 could potentially play a role in dopaminergic neuron viability and in PD.

## Introduction

Parkinson’s disease (PD) is characterized by degeneration of dopaminergic neurons in substantia nigra pars compacta by mechanisms that are not fully understood (Jenner and Olanow 2006; Gupta et al. 2008; Lees et al. 2009). Mitochondrial dysfunctions and changes in cell metabolism with an altered growth factor signaling are associated with the disease process in PD (Abou-Sleiman et al. 2006; Lin and Beal 2006; Banerjee et al. 2009). Mitochondria influence a number of cellular functions ranging from regulation of cell energy and metabolism, and the control of intracellular calcium and cell death pathways (Lindholm et al. 2004; Nunnari and Suomalainen 2012). Mitochondria are also the major source of reactive oxygen species (ROS) in the cell occurring as a consequence of oxidative phosphorylation. In neurodegenerative disease including PD increased levels of ROS are thought to contribute to neuronal loss (Henchcliffe and Beal 2008; Zhou et al. 2008).

Peroxisome proliferator activated receptor γ coactivator 1α (PGC-1α) is a transcriptional coactivator that is a master regulator of mitochondrial biogenesis and cell viability (Wu et al. 1999; Houten and Auwerx 2004; Lin et al. 2005). PGC-1α is also involved in cell defense against oxidative stress by stimulating production of antioxidant enzymes in the cell (St-Pierre et al. 2006). PGC-1α has been studied mainly for its action in cell and lipid metabolism and in metabolic disease such as diabetes and obesity (Handschin and Spiegelman 2006; Kleiner et al. 2012). In the brain gene deletion of PGC-1α caused an increased susceptibility of neurons against excitotoxic injury (St-Pierre et al. 2006; Cui et al. 2006). A meta-analyses of PD patient samples indicated that PGC-1α and its gene networks are potential therapeutic targets for early intervention in the disease (Zheng et al. 2010). Recent studies with overexpression of PGC-1α in mouse brain showed that it exerts a protective effect on dopaminergic neurons in the 1-methyl-4-phenyl-1, 2, 3, 6-tetrahydropyridine (MPTP) mouse model of PD (Mudo et al. 2012).

Available data shows that PGC-1α is regulated by both transcriptional and posttranscriptional mechanisms (Houten and Auwerx 2004; Lindholm et al. 2012). The deacetylase Sirtuin-1 (SIRT1) can activate PGC-1α by means of protein deacetylation as shown in different cell types (Rodgers and Puigserver 2007; Nemoto et al. 2005). SIRT1 is a NAD^+^-dependent enzyme that plays a critical role in the regulation of metabolism and the cell response to nutrients and caloric restriction (Rodgers and Puigserver 2007; Revollo et al. 2004; Lagouge et al. 2006; Alcaín and Villalba 2009). Changes in SIRT1 has been linked to aging (Alcaín and Villalba 2009; Mercken et al. 2013) and to metabolic and neurodegenerative diseases (Mudo et al. 2012; Kim et al. 2007; Outeiro et al. 2008).

Fibroblast growth factor-21 (FGF21) a member of the fibroblast growth factor 19 subfamily together with FGF19 and FGF23 and is primarily expressed in the liver (Nishimura et al. 2000). FGF21 has an endocrine function in the body and plays a role in cell metabolism by stimulating glucose uptake (Kharitonenkov et al. 2005) by influencing fatty acid metabolism (Potthoff et al. 2009) and by controlling lipoprotein receptor (LDLR) levels and lipoprotein uptake in liver cells (Do et al. 2012). FGF21 was shown to increase PGC-1α in liver and fat tissue (Potthoff et al. 2009; Fisher et al. 2012) and to regulate energy homeostasis in adipocytes via the SIRT1-PGC1 pathway (Chau et al. 2010). The expression and functions of FGF21 in brain cells are so far unknown.

In this work, we have studied the role of FGF21 in the regulation of PGC-1α and mitochondria in human dopaminergic neurons. As a model we employed cultured human dopaminergic neurons derived from midbrain neuronal precursor cells obtained from human embryo (Lotharius et al. 2002). Results showed that FGF21 influences the levels and activity of PGC-1α in the human dopaminergic neurons by increasing SIRT1 and nicotinamide adenine dinucleotide (NAD^+^) in the cells. The activation of PGC-1α was followed by increased levels of the antioxidant enzymes, sodium dismutase 2 (SOD2) and thioredoxin 2 (Trx2) and by an enhanced mitochondrial respiratory capacity as revealed by real-time in living neurons.

## Materials and methods

### Cell culture

Human mesencephalon neuronal precursor cells (MESC2.10 cells) were cultured in poly-D-lysine (Sigma, St Louis, MO, USA) coated flasks (75 cm^2^) in Dulbeccos modified Eagle medium (DMEM)/F12 medium (Gibco, Invitrogen, Calrsbad, CA, USA) supplemented with B27 (Gibco) and Penicillin/streptomycin and human basic FGF 20 ng/ml (Peprotech, Rocky Hill, NJ, USA). To induce neuronal differentiation, cells were cultured for 6 days on poly-D-lysine/laminin (Sigma) coated wells at a density of 30,000 cells /cm^2^ in a medium containing 1 μg/ml tetracyclin (Sigma) (Lotharius et al. 2002; Di Liberto et al. 2012). Medium was changed every second day and cell differentiation was monitored by the expression of markers for dopaminergic neurons such as tyrosine hydroxylase and dopamine transporter. Human dopaminergic cells were treated with 50 ng/ml FGF21 and analyzed as described in the text. In some experiments 20 μM nicotinamide (NAM) was used to the cells to inhibit SIRT1. Glial cultures from newborn rodent brains were prepared and cultured in 10% fetal calf serum in DMEM as described previously (Lindholm et al. 1992; Mäkelä et al. 2010). The human hepatocyte cell line (Huh7) was used as a positive control for FGF21 expression (Do et al. 2012).

### Immunoblotting

Differentiated MESC2.10 cells were treated with 50 ng/ml FGF21 (R&D Systems, Minneapolis, MN, USA) for 24 h and cells were lysed in RIPA buffer containing 150 mM NaCl, 1% NP-40, 0,25% sodium deoxycholate, 50 mM Tris-HCl pH 7.4, and 0,1% sodium dodecyl sulfate (SDS). Immunoblotting was done essentially as described (Mudo et al. 2012; Do et al. 2012; Korhonen et al. 2001; Sokka et al. 2007). In brief, 30 μg of protein was run on SDS-PAGE and transferred to Hybond-C Extra nitrocellulose membrane (Amersham Biosciences, Buckinghamshire, UK). The membrane was blocked in 5% non-fat milk-Tris buffered saline (TBS) for 1 h and room temperature and primary antibodies were added overnight at +4˚C. The antibodies used were: anti-SIRT1 (diluted 1:1000; Cell Signaling Technology, Danvers, MA, USA), anti-PBEF/NAMPT (1:1000; Abcam, Cambridge, UK), anti-SOD2 (1:5000; AbFrontier, Seoul, Korea), anti-Trx2 (1:1000; AbFrontier), anti- PGC-1α (1:5000; Calbiochem, San Diego, CA, USA), anti- cytochrome oxidase IV (COX IV, 1:2000; Abcam), anti-mitochondrial transcription factor A (TFAM, 1:1000; Abcam), anti-FGF21 (1:3000; Novus Biologicals, Littleton, CO, USA) and anti-β-actin (1:5000; Sigma). The membranes were washed with TBS-5% Tween20 buffer and appropriate horseradish peroxidase (HRP)-conjugated secondary antibodies (1:2500; Jackson ImmunoResearch, West Grove, PA, USA) were added for 1 h at room temperature. Super Signal West Pico chemiluminescent substrate (Thermo, Waltham, MA, USA) was used for visualization of the bands, and ImageJ software for their quantification.

Different brain regions of adult mouse were dissected under stereomicroscopy and frozen in cooled isopentane as described before (Mudo et al. 2012). Tissue pieces were homogenized in cold RIPA buffer containing also 10 μg/ml aprotinin, 10 μg/ml leupeptin, 0.1 mM phenylmethylsulfonyl fluoride, 100 μM sodium orthovanadate (Na_3_VO_4_) and 1 mM ethylenediaminetetraacetic acid (EDTA; Sigma-Aldrich, St. Louis, MO, USA). The homogenate was left on ice for 30 min and centrifuged at 10,000 × g for 15 min at 4°C to yield supernatant fractions that were stored at−80°C until use. To obtain enough material, the two SN were pooled. 30-40 μg of protein was subjected to immunoblotting as above using primary antibodies anti-FGF21 (1:3000) and β-actin as control. The membrane was processed as above using secondary antibodies and visualisation of bands was made by enhanced chemiluminescence.

### Immunoprecipitation

Experiments were done essentially as described (Mudo et al. 2012; Revollo et al. 2004) using lysates from human dopaminergic cells treated with 50 ng/ml FGF21 for 24 h alone or together with 10 mM nicotinamide (NAM; Acros organics, Geel, Belgium). In brief, 1,5 μg of anti- PGC-1α antibody (Calbiochem) was added to 500 μg of cell lysates overnight at +4˚C under constant rotation. 50 μl of Protein G-agarose (Roche, Basel, Switzerland) was then added to the samples and incubated for 6 h. Beads were washed three times with RIPA buffer and samples were run on 8% SDS-PAGE, followed by transfer and immunoblottings as described above using first anti-acetylated lysine antibodies (1:1000; Cell signaling technology) and then anti-PGC-1α antibodies. The quantifications of the bands were done using ImageJ software. The degree of acetylation of PGC-1α is an index for the activity of this protein in the cell (Mudo et al. 2012; Rodgers and Puigserver 2007).

### Luciferase assay

Cells were transfected with 0,5 μg of the PGC-1α–luciferase reporter constructs or the control pGL3 promoter constructs together with 0,025 μg Renilla pRL-TK plasmid using Fugene (Promega, Madison, WI, USA). Cells were incubated for 2 days in the differentiation medium and 50 ng/ml FGF21 was added for an additional 24 h. Luciferase activity was measured using the Dual-Luciferase reporter Assay (Promega) and a GLOMAX 20/20 luminometer (Promega) as described previously (Mudo et al. 2012; Kairisalo et al. 2009). The values were normalized to those of Renilla.

### Determination of NAD^+^/NADH levels

To analyze NAD^+^/NADH level in cells the absorbance at 450 nm was measured using Multiscan MS Version 3.0 spectrophotometer following the assay kit as provided by the manufacturer (Abcam).

### Real-time analyses of mitochondrial respiratory capacity in human dopaminergic neurons

Cells were plated on Seahorse 96well plate (Seahorse Bioscience, Boston, MA USA) and differentiated for 5 days followed by a 24 h-stimulation with 50 ng/ml FGF21. Medium was changed to HCO_3_-free DMEM (Sigma) containing 10 mM pyruvate, 1 mM l-glutamine and 10 mM glucose 1 h prior to analyses and keeping cells at +37˚C without CO_2_. Oxygen consumption rate (OCR) in dopaminergic neurons was determined in real-time using theSeahorse XFe96 analyzer (Seahorse Bioscience) by determining oxygen consumption as response to the addition of various chemicals. Three 3 min cycles were run for every measurement and the Mitostress kit (Seahorse) was used containing the following compounds: 1 μM Oligomycin (ATP synthase inhibitor), 0,8 μM Carbonyl cyanide 4-(trifluoromethoxy)phenylhydrazone (FCCP) (mitochondrial uncoupler), 1 μM Rotenone (complex I inhibitor of the respiratory chain) and Antimycin A (complex III inhibitor).

### Mitochondrial DNA copy number

DNA was isolated from human dopaminergic neurons using QIAamp DNA Mini Kit (QIAGEN, Hilden, Germany) according to manufacturer’s protocol. The relative mitochondrial DNA (mtDNA) copy number was measured using quantitative PCR and comparing the ratio of mtDNA to nuclear DNA and the method described previously (Xie et al. 2013). In brief, the mitochondrially encoded *NADH dehydrogenase 1 (ND1 gen*e*)* was used for quantifying mtDNA and human *globulin (HGB) gene* for nuclear DNA. The primer sequences used were: forward primer ND1-F: 5′-CCT AAA ACC CGC CAC ATC T-3′, reverse primer ND1-R: 5′-GAG CGA TGG TGA GAG CTA AGG T-3′; forward primer HGB-F: 5′-GTG CAC CTG ACT CCT GAG GAG A-3′, and reverse primer HGB-R: 5′-CCT TGA TAC CAA CCT GCC CAG-3′. The PCR reaction mixture contained 5 ng DNA and 200 nmol/l of forward and reverse primers in 1X SYBR Green Mastermix (Roche). The reaction was carried out at 95˚C for 10 min followed by at 95˚C for 15 s and 60˚C for 1 min using 40 cycles. Each sample was run in triplicates on a 96-well plate and water was used as negative control. Quantitative-PCR (qPCR) amplification was performed using Light Cycler 480 II instrument (Roche) as described (Do et al. 2012; Hyrskyluoto et al. 2013). The ratio of mtDNA to nuclear DNA reflects the concentration of mtDNA per dopaminergic cell.

### Electron microscopy and mitochondrial density analysis

Human dopaminergic neurons were stimulated with 50 ng/ml FGF21 for 24 h, fixed with 2,5% glutaraldehyde in PBS for 1 h at room temperature, and washed two times for 1 h with H_2_O. Postfixation was done using 1% osmiumtetroxide. The embedding and sectioning procedures were done essentially as described before (Korhonen et al. 2008). The sections were stained with lead citrate and uranyl acetate and viewed using a Jeol JEM-1400 transmission electron microscope (Jeol Ltd., Tokyo, Japan) equipped with Gatan Orius SC 1000B bottom mounted CCD-camera (Gatan Inc., USA). Mitochondria identified in the EM pictures of the cells were marked manually and the relative surface area was calculated using the ImageJ software. Results were pooled from different sections of control and FGF21-treated cells and the density of mitochondria was compared.

### Quantification and statistics

Immunoblots were quantified with ImageJ quantification software. Results are expressed as percentage of controls and statistical analyses were performed using one-way analysis of variance (ANOVA) and Bonferroni’s multiple comparison tests. Values are given as means ± SD and p < 0.05 was considered as statistically significant.

## Results

### FGF21 increases levels and deacetylation of PGC-1α in cultured human dopaminergic neurons

**Figure 1:**
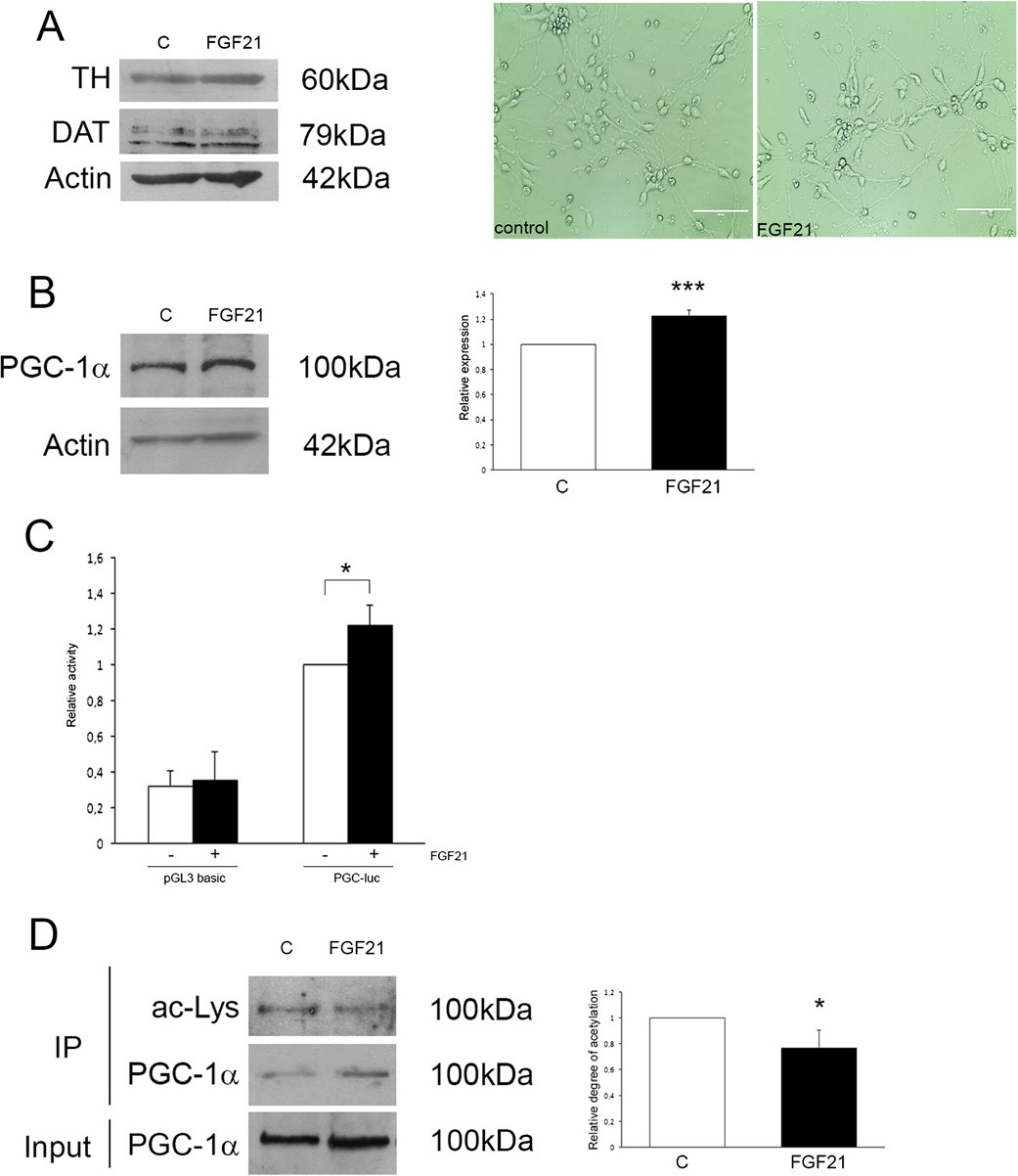
**Effect of FGF21 on the expression and activation of PGC-1α in human dopaminergic neuron.** Human dopaminergic neurons differentiated from midbrain progenitor cells were cultivated as described in Materials and methods. Cells were treated with 50 ng/ml FGF21 for 24 h as indicated below. **(A)** Left, immunoblot showing expression of tyrosine hydroxylase (TH) and dopamine transporter (DAT) in the human dopaminergic cells. β-actin was used as control. Right, phase contrast pictures. There was no significant difference in morphology between control and FGF21 treated cells. Scale bar. **(B)** Left, immunoblot. β-actin was used as control. The levels of PGC-1α were increased by FGF21. Right, quantification was done using ImageJ software. Values are means ± SD, n = 4, ***p < 0.001 for FGF21 vs C. **(C)** Gene promoter assays. Cells were transfected with the pGL3 basic plasmid and the PGC-1α promoter plasmids linked to a luciferase reporter. Cells were stimulated with FGF21 for 24 h and the luciferase activity was measured and corrected for that of *Renilla* as described as described in Methods. FGF21 enhanced *PGC-1α gene* activity but not that of the control pGL3 promoter. Values are means ± SD, n = 4, *p < 0.05 for FGF21 vs controls. **(D)** Immunoprecipitation experiments. PGC-1α was immunoprecipitated from control and FGF21-treated cells followed by immunoblotting as described in Methods. The degree of acetylation of PGC-1α was analyzed using the anti-acetylated lysine antibody. Total amount of PGC-1α in the immunoprecipitate was analyzed using anti- PGC-1α antibodies. Values are means ± SD, n = 4, *p < 0.05 for FGF21 vs C.

In this work we used midbrain progenitor (MESC2.10) cells from human embryonic brain (Lotharius et al. 2002) that were further differentiated into dopaminergic neurons in culture (Figure 1A). Treatment of the cells with 50 ng/ml FGF21 for 24 h led to an increase in PGC-1α as shown by immunoblotting (Figure 1B). To clarify whether this involved increased gene expression we used the *PGC-1α*-promoter linked to a luciferase reporter gene. Data showed that the gene activity of the PGC-1α promoter was increased by FGF21 with no changes in the activity of the pGL3 basic-promoter used as control (Figure 1C). There was also an increase in PGC-1α mRNA levels in FGF21-treated dopaminergic neurons using quantitative PCR (data not shown). To study whether FGF21 may activate PGC-1α we performed immunoprecipitation experiments followed by immunoblotting using anti-PGC-1α and anti-acetylated lysine antibodies. Data obtained showed that the relative degree of acetylation of PGC-1α decreased in cells treated with FGF21 as compared to control (Figure 1D). Collectively these data indicate that FGF21 can increase both the level and the activation status of PGC-1α in the human dopaminergic neurons.

### FGF21 increases Nampt and NAD^+^ levels and SIRT1 in the dopaminergic neurons

**Figure 2:**
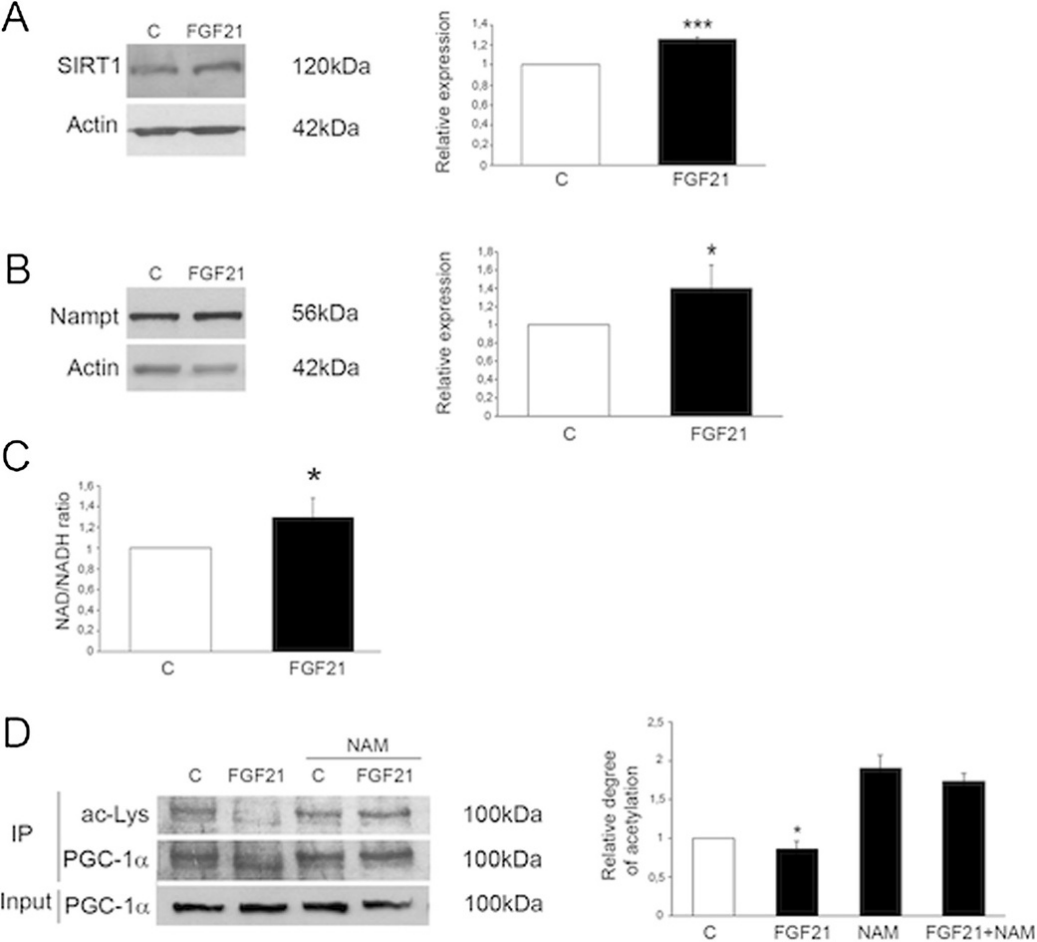
**FGF21 increases SIRT1 and Nampt and the level of NAD** ^**+**^ **in human dopaminergic neurons.** Human dopaminergic neurons were treated with 50 ng/ml FGF21 for 24 h as indicated below. **(A-B)** Immunoblots. β-actin was used as a control. The levels of SIRT1 **(A)** and Nampt **(B)** were increased by FGF21. Left, immunoblots. Right, quantifications using ImageJ. Values are means ± SD, n = 4, ***p < 0.001 and *p < 0.05 for FGF21-treated vs C. **(C)** The ratio of NAD^+^ to NADH in the dopaminergic neurons was measured as described in Methods. FGF21 increased NAD^+^ levels in the neurons. Values are means ± SD, n = 4, *p < 0.05 for FGF21 vs C. **(D)** Immunoprecipitation experiments. Cells were stimulated with FGF21 for 24 h in the absence or presence of 20 μM NAM to inhibit SIRT1. The degree of acetylation of PGC-1α was analyzed as above. Left, immunoblot. Right, quantification. FGF21 decreased PGC-1α acetylation in control cells but not in NAM treated cells. There was also an increase in acetylated PGC-1α due to SIRT1 inhibition as compared with controls. Values are means ± SD, n = 4, *p < 0.05 for FGF21 vs C.

PGC-1α can be post-transcriptionally modified in cells that affect its properties as gene coactivator (Houten and Auwerx 2004; Lin et al. 2005). SIRT1 is known to deacetylate PGC-1α increasing its activity (Rodgers and Puigserver 2007). We were therefore interested to study possible effects of FGF21 on SIRT1 in the human dopaminergic neurons. Data showed that stimulation of cells for 24 h with 50 ng/ml FGF21 increased the levels of SIRT1 as analyzed by western blot (Figure 2A). We also observed that FGF21 treatment increased the levels of Nampt, which is the rate-limiting enzyme in NAD^+^ biosynthesis (Figure 2B). FGF21 also increased the NAD^+^ levels in the dopaminergic neurons as shown using the NAD^+^/NADH assay (Figure 2C). This shows that FGF21 increases Nampt and the NAD^+^ levels in the dopaminergic neurons that subsequently activate SIRT1 and PGC-1α in these cells. To substantiate this further, we incubated the neurons in the presence of the SIRT1-inhibitor NAM that increased the degree of acetylation of PGC-1α in the cells (Figure 2D). NAM also counteracted the deacetylation of PGC-1α induced by FGF21 in these neurons (Figure 2D). Collectively these results show that FGF21 influences the Nampt/SIRT1 pathway in the human dopaminergic neurons leading to the activation of PGC-1α.

### FGF21 increases mitochondrial antioxidants in human dopaminergic neurons

**Figure 3:**
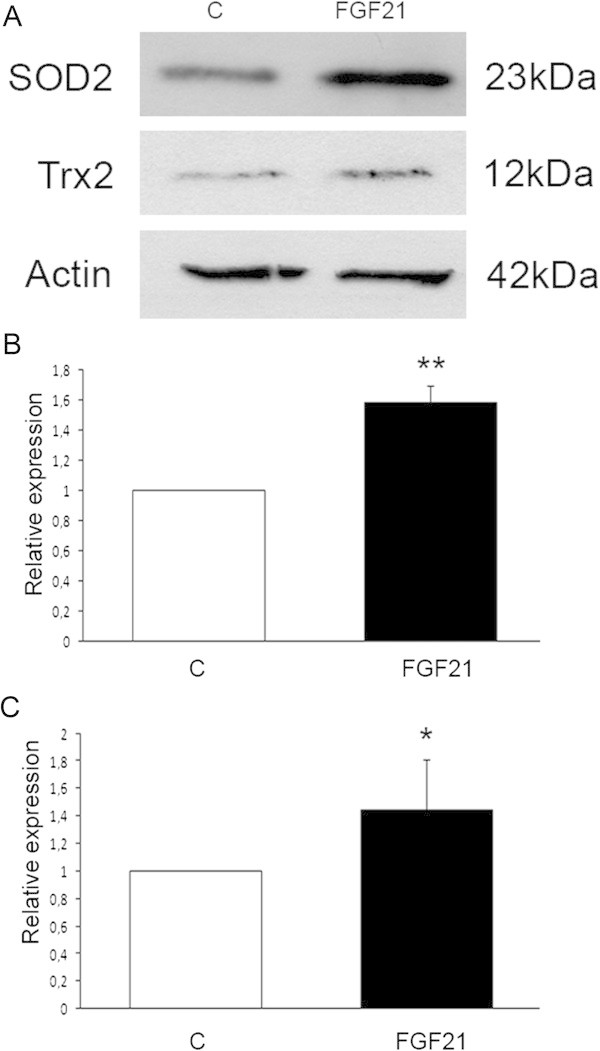
**FGF21 elevates mitochondrial antioxidants in the dopaminergic neurons.** Human dopaminergic neurons were treated with 50 ng/ml FGF21 for 24 h followed by immunoblotting. **(A)** Immunoblots. β-actin was used as a control. FGF21 increased the levels Sod2 and Trx2 that are antioxidants localized to mitochondria. **(B-C)** Quantification. Sod2 **(B)** and Trx2 **(C)** increased in FGF21-treated neurons. Values are means ± SD, n = 4, **p < 0.01 or *p < 0.05 for FGF21 vs C.

We have previously shown that overexpression of PGC-1α in transgenic mice led to an increase in the levels of SOD2 and Trx2, two mitochondrial antioxidants, in the substantia nigra harboring the dopaminergic neurons (Mudo et al. 2012). We therefore studied whether these antioxidants were also increased in human dopaminergic neurons after FGF21 treatments. Data showed that SOD2 and Trx2 were both significantly upregulated by FGF21 in these neurons (Figure 3A-C). This suggests that FGF21 via activation of PGC-1α can increase these mitochondrial antioxidants that may contribute to neuroprotection against oxidative stress.

### FGF21 stimulates mitochondrial respiratory capacity of human dopaminergic neurons

**Figure 4:**
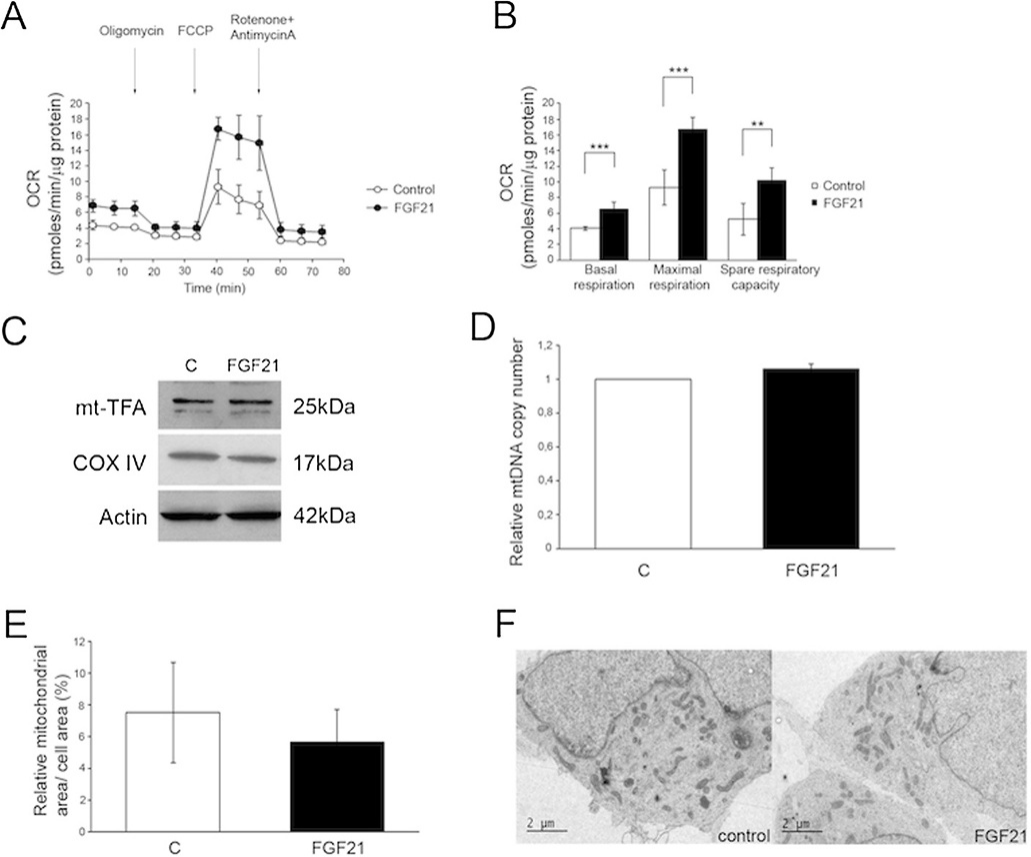
**Effect of FGF21 on the mitochondria respiratory capacity and mitochondria number in human dopaminergic neurons.** Human dopaminergic neurons were stimulated with 50 ng/ml FGF21 and analyzed further as indicated below. **(A)** Oxygen consumption rate (OCR) in human dopaminergic neurons was measured in real-time using the Seahorse equipment as described in Methods. Typical graphs after addition of the various inhibitors are shown. **(B)** Quantification. Basal and maximum respiration (OCR) were increase in cells treated with FGF21 as was the spare respiratory capacity. Values are means ± SD, n = 4. ***p < 0.001 for FGF21 vs C. **(C)** Immunoblots. β-actin was used as control. The mitochondrial proteins TFAM and COX IV showed no significant changed in dopaminergic neurons treated with FGF21. **(D)** The mitochondrial DNA (mtDNA) copy number was analyzed using quantitative PCR as described in Methods. There was no change in the relative ratio of mtDNA to nuclear DNA reflecting an equal number of mitochondria in control and FGF21-treated dopaminergic cells. **(E-F)** Mitochondrial surface area. Left, control and FGF21 treated cells were analyzed by electron microscopy and the relative mitochondrial area was calculated from EM pictures as described in Methods. There were no significant changes in the mitochondrial surface area between control and FGF21 treated cells. Values are means means ± SD, n = 4. Right, typical EM pictures of control and FGF treated cells.

To study the effect of FGF21 on mitochondrial functions in dopaminergic neurons further we analyzed the cells in real-time using the Seahorse equipment as described in Methods. Data showed that the human dopaminergic neurons stimulated with FGF21 had an increased basal respiration and a higher maximal respiratory capacity as compared to control cells (Figure 4A-B). This result together with data on the antioxidants indicates that FGF21 is able to increase the mitochondrial efficacy and cell resistance towards oxidative stress in human dopaminergic neurons. We were also interested to investigate whether the number and biogenesis of mitochondria could be altered by FGF21 in the cultured human dopaminergic cells. To clarify this we first analyzed the levels of TFAM and of COX IV as markers for mitochondria. Data showed that neither of these proteins was increased by a 24 h-FGF21 treatment of dopaminergic neurons compared with controls (Figure 4C). Estimation of the mtDNA content using qPCR revealed also no change in the ratio of mtDNA to nuclear DNA after FGF21 stimulations indicating an equal number of organelles in control and FGF21-treated dopaminergic neurons (Figure 4D). To substantiate this we also studied the morphology of mitochondria using electron microscopy (EM) (see Methods). Measurements of the relative mitochondrial area from EM sections showed no significant change between control and FGF21-treated cells (Figure 4E-F). Taken together these results show that FGF21 can increase the efficacy of the mitochondria but that this is not related to an increased mitochondria number in the dopaminergic cells after treatment with FGF21.

### FGF21 is expressed in the midbrain and by glia cells in culture

**Figure 5:**
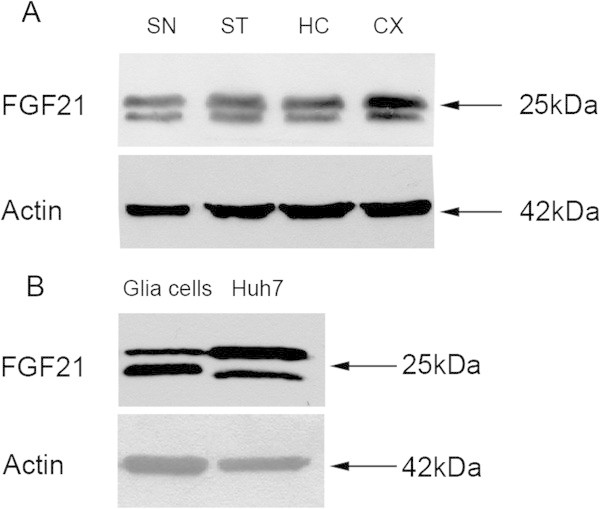
**FGF21 is expressed in brain tissue and by glia cells in culture. (A)** Brain regions from adult mice were dissected and subjected to immunoblotting using anti-FGF21 antibodies. β-actin was used as control. FGG21 is present in all brain areas. Two bands are detected representing the precursor and processed, mature form of FGF21 respectively. SN, substantia nigra; ST, striatum; HC, Hippocampus; CX, Cortex. **(B)** Immunoblot. Glia cell cultures were made from neonatal rat brain as described in Methods. Immunoblotting of cell lysates were performed using anti-FGF21 antibodies. The human hepatocyte cell line (Huh7) was used as a positive control. Note the presence of FGF21 in both cell types.

To study whether FGF21 is present in brain tissue, we performed immunoblotting experiment using samples from adult rodent brain and antibodies against FGF21. Data showed that FGF21 is present in different brain regions including substantia nigra and striatum that contain the cell bodies and the terminals of the midbrain dopaminergic neurons respectively (Figure 5A). Studies *in vitro* showed that primary glial cultures obtained from neonatal rat brain expressed FGF21 as shown by immunoblotting (Figure 5B). The relative levels of FGF21 expressed by these cells correspond to those found in the human hepatocyte Huh7 cell line (Figure 5B). This data suggest that glial cells can produce FGF21 as studied *in vitro.* Immunohistochemical analyses employing the anti-FGF21 antibody were not conclusive, and the precise localization of FGF21 in different brain cells will require more studies in the future.

## Discussion

FGF21 is a growth factor that has been studied mainly for its effects on metabolism and cell responses in peripheral cells. The present work demonstrates that FGF21 can increase both the mitochondrial respiratory capacity and PGC-1α in human dopaminergic neurons. These two effects of FGF21 in the human dopaminergic neurons are probably interconnected and related to the activation of PGC-1α by this growth factor. PGC-1α is known to be a major regulator of mitochondria biogenesis and functions via regulation of gene expression (Houten and Auwerx 2004; Lin et al. 2005). PGC-1α also takes part in protection against cell stress and oxidative damage accompanying human metabolic disorders and degenerative diseases (St-Pierre et al. 2006). In line with this we found that FGF21 increased the levels of the antioxidant enzymes SOD2 and Trx2 localized to mitochondria in the human dopaminergic neurons. Previous studies using transgenic mice overexpressing PGC-1α in dopaminergic neurons also showed an increase in these antioxidants in the midbrain. Furthermore, the compound resveratrol acting via the SIRT1/ PGC-1α can also stimulate SOD2 and Trx2 levels both in cultured neurons and in the brain (Mudo et al. 2012; Kairisalo et al. 2011). As shown *in vivo*, the increase in mitochondrial antioxidants by PGC-1α was accompanied by cell protection of dopaminergic neurons against the neurotoxin MPTP-induced oxidative stress (Mudo et al. 2012). Together these results show that PGC-1α is an important factor in regulation of dopaminergic neuron viability and that growth factors like FGF21 may act via the induction of PGC-1α and its downstream pathways in the neurons.

The mechanisms by which PGC-1α are regulated have been studied in different cell types and shown to be rather complex (Houten and Auwerx 2004; Lindholm et al. 2012). Available data indicates that both transcriptional and post-transcriptional events are involved in the control of this protein. In this work we studied the mechanism by which FGF21 influences PGC-1α in neurons using human dopaminergic cells as a model system (Lotharius et al. 2002; Di Liberto et al. 2012). Data showed that FGF21 increased the regulatory protein SIRT1 with the ability to de-acetylate and activate PGC-1α. NAD^+^ levels are crucial in the control of biosynthetic reactions and cell metabolism that occurs partly via SIRT1. We observed that FGF21 elevated NAD^+^ in the dopaminergic neurons following an increase in the enzyme Nampt that is rate limiting in the biosynthesis of NAD^+^ from NAM (Revollo et al. 2004; Yang et al. 2006). These results show that FGF21 can affect SIRT1 activity via an increase in the Nampt/NAD levels in the dopaminergic neurons. Previous studies have shown that the expression of Nampt also called pre-B-cell colony-enhancing factor (PBEF) or visfatin can be induced by nutrient restriction and by cytokines (Yang et al. 2006; Wang et al. 2006). These studies have been concerned mainly with fibroblast, vascular smooth cells or with pancreatic β-cells. In this work we show that the Nampt-SIRT1 pathway is also active in neurons and can be regulated by FGF21 signaling. These results in dopaminergic neurons add to previous data showing an involvement of Nampt in the regulation of metabolic responses, inflammation, and cell differentiation in peripheral cells (Garten et al. 2009). Given its role in the activation of SIRT1 in dopaminergic neurons it would be interesting to study the regulation of Nampt in animal models of PD and after treatment with other factors.

To prove the point that FGF21 can stimulate mitochondrial functions in the dopaminergic neurons we performed a real-time analysis of the cells using the Seahorse equipment. Data showed that FGF21 is able to increase the mitochondrial respiratory capacity of the human dopaminergic neurons. Though not formally proven, we suspect that this effect is due to the activation of PGC-1α by FGF21 in these neurons. This data made in neurons is in line with previous results on the regulation of mitochondrial functions by the SIRT1/ PGC-1α in muscle cells (Gerhart-Hines et al. 2007). Furthermore, we here also analyzed whether the copy number of these organelles increased by this growth factor. For this we studied the mitochondrial DNA copy number using PCR, and the expression of TFAM a regulator of mitochondrial biogenesis proteins and COX IV as a protein of the respiratory chain. Together the results showed that there was no significant change in any of these parameters following FGF21 treatment. This suggests that the copy number is not altered by FGF21 and that the respiratory capacity is mainly increased in the dopaminergic neurons by this growth factor. A constant level of mitochondria may arise from changes in biogenesis and degradation of the organelle that balance each other. In the future it will be interesting to study whether FGF21 may also affect the dynamics of mitochondria in dopaminergic neurons.

Previous studies have shown that several members of the large fibroblast growth factor (FGF) gene family are also expressed in the central nervous system (Itoh and Ornitz 2011). The FGFs have important functions both during development and in the mature brain (Itoh and Ornitz 2011; Mudò et al. 2009; Lahti et al. 2012). So far little is known about the expression of FGF21 in the brain. Using immunoblots we show here that FGF21 is expressed in various brain regions of adult mouse to an appreciable amount. Studies on primary brain cell cultures showed that glial cells can produce FGF21 in substantial amounts at least *in vitro*. Immunohistochemical analyses using the FGF-21 antibodies proven hard to perform and gave ambiguous results. Further studies using other antibodies or *in situ* hybridization experiments are therefore required to determine which cell types specifically express FGF21 in the brain.

One of the issues not addressed in this study is the nature of the receptors mediating the effects of FGF21 on human dopaminergic neurons. There are four types of FGF receptors, and FGF21 was shown to interact with at least FGF receptors 1-3 (Suzuki et al. 2008; Kharitonenkov et al. 2008). In addition, the protein β-Klotho is required for FGF21 to exert its biological actions (Suzuki et al. 2008; Kharitonenkov et al. 2008) although this may not be the case for all types of cells (Tomiyama et al. 2010). Previous studies using in situ hybridization revealed an expression of Fibroblast growth factor receptor 1 (FGFR1) in substantia nigra neurons in adult rats (Belluardo et al. 1997). However fewer data are available on human neurons, and the receptors by which FGF21 stimulates human dopaminergic neurons warrant further studies in the future.

The present study showing effects of FGF21 in dopaminergic neurons may have further physiological relevance for the regulation of cell signaling events and metabolism in brain cells. It has been proposed that neurodegeneration is accompanied by changes in neuronal metabolism or dysfunctional cell signaling that may be alleviated by the use of various drugs or growth factors (Patrone et al. 2013). FGF21 is a metabolic regulator has many beneficial effects on cell metabolism and in human metabolic diseases. The expression of FGF21 can be induced by prolonged fasting (Kharitonenkov et al. 2005; Inagaki et al. 2007) and FGF21 may also penetrate into brain tissue (Hsuchou et al. 2007). It was recently also shown using transgenic mouse that FGF21 is able to increase the life span of the animals probably due to altered cell signaling cascades (Zhang et al. 2012).

In conclusion, the present study shows that dopaminergic neurons respond to FGF21 by enhancing the mitochondrial capacity and altering gene pathways regulated by PGC-1α in these cells. These results suggest that FGF21 may be of value in considering neuroprotection strategies for PD. In the future it would be interesting to study the potential benefits can be afforded by FGF21 in different neurological disorders.

## Abbreviations

ANOVA: One-way analysis of variance
COX IV: Cytochrome oxidase IV
DMEM/F12: Dulbeccos modified Eagle/F12 medium
EM: Electron microscopy
FCCP: Carbonyl cyanide 4-(trifluoromethoxy)phenylhydrazone
FGF: Fibroblast growth factor gene family
FGF21: Fibroblast growth factor-21
FGFR1: Fibroblast growth factor receptor-1
mtDNA: Mitochondrial DNA
Nampt: Nicotinamide phosphoribosyltransferase
OCR: Oxygen consumption rate
PBEF: Pre-B-cell colony-enhancing factor
PD: Parkinson’s disease
PGC-1 α: Peroxisome proliferator-activated receptor γ coactivator-1 α
SIRT1: Sirtuin-1
qPCR: Quantitative-PCR.
